# Haptoglobin gene subtypes in three Brazilian population groups of different ethnicities

**DOI:** 10.1590/S1415-47572009005000051

**Published:** 2009-09-01

**Authors:** Ana L. Miranda-Vilela, Arthur K. Akimoto, Penha C. Z. Alves, Cássia O. Hiragi, Guilherme C. Penalva, Silviene F. Oliveira, Cesar K. Grisolia, Maria N. Klautau-Guimarães

**Affiliations:** Departamento de Genética e Morfologia, Instituto de Ciências Biológicas, Universidade de Brasília, Brasília, DFBrazil

**Keywords:** Brazilian ethnicities, haptoglobin, polymorphism, subtypes

## Abstract

Haptoglobin is a plasma hemoglobin-binding protein that limits iron loss during normal erythrocyte turnover and hemolysis, thereby preventing oxidative damage mediated by iron excess in the circulation. Haptoglobin polymorphism in humans, characterized by the Hp^*1^ and Hp ^*2^ alleles, results in distinct phenotypes known as Hp1-1, Hp2-1 and Hp2-2, whose frequencies vary according to the ethnic origin of the population. The Hp^*1^ allele has two subtypes, Hp ^*1F^ and Hp ^*1S^ , that also vary in their frequencies among populations worldwide. In this work, we examined the distribution frequencies of haptoglobin subtypes in three Brazilian population groups of different ethnicities. The haptoglobin genotypes of Kayabi Amerindians (n = 56), Kalunga Afro-descendants (n = 70) and an urban population (n = 132) were determined by allele-specific PCR. The Hp^*1F^ allele frequency was highest in Kalunga (29.3%) and lowest in Kayabi (2.6%). The Hp^*1F^/Hp^*1S^ allele frequency ratios were 0.6, 1.0 and 0.26 for the Kayabi, Kalunga and urban populations, respectively. This variation was attributable largely to the Hp^*1F^ allele. However, despite the large variation in Hp^*1F^ frequencies, results of F _ST_ (0.0291) indicated slight genetic differentiation among subpopulations of the general Brazilian population studied here. This is the first Brazilian report of variations in the Hp^*1F^ and Hp^*1S^ frequencies among non-Amerindian Brazilians.

## Introduction

Molecular variation in human haptoglobin (Hp) was described by Smithies (1955), who identified three major phenotypes, Hp1-1, Hp2-1, and Hp2-2, by starch gel electrophoresis. These phenotypes are controlled by two autosomal codominant alleles, Hp^*1^ and Hp^*2^ (Smithies and Walker, 1956). Two subtypes of the Hp^*1^ allele, Hp^*1S^ and Hp^*1F^, were subsequently identified in urea-containing starch gels (Smithies *et al.*, 1962). Despite populational differences in the distribution of haptoglobin phenotypes, these alleles have been found in every human population examined so far. The Hp^*1^ allele frequency is lower in South-East Asia and higher in South America (Langlois and Delanghe, 1996). There is also a significant difference in the frequency distribution of Hp^*1F^ and Hp^*1S^ alleles among populations worldwide (Carter and Worwood, 2007). An extreme case of populational variation was reported for the Hp^*1F^ allele in which a geographical cline of this allele increased in the same direction as the Hp^*1^ allele, whereas the Hp^*1S^ frequency showed no variation (Delanghe *et al.*, 2000).

The Hp phenotypes determine the serum levels of Hp-glycoprotein but differ in their number of protein components, electrophoretic mobility, plasma concentration of Hp, and antioxidant and antiinflammatory activities, often with divergent clinical consequences (Langlois and Delanghe, 1996; Yano *et al.*, 1998; Wassel, 2000; Koch *et al.*, 2003; Sadrzadeh and Bozorgmehr, 2004; Tseng *et al.*, 2004; Carter and Worwood, 2007). Many clinical studies have demonstrated a link between Hp polymorphism and a broad range of pathological conditions, and such associations probably reflect functional differences among the phenotypes (Langlois and Delanghe, 1996; Wassel, 2000; Sadrzadeh and Bozorgmehr, 2004; Levy, 2006; Zvi and Levy, 2006). In contrast, other studies have reported no such associations, despite the wide range of Hp*^1^ and Hp*^2^ gene frequencies throughout the world (Carter and Worwood, 2007). These apparently divergent findings can only be understood through additional characterization of the distribution of Hp subtype polymorphisms.

The Brazilian population is very mixed, primarily as a result of five centuries of interethnic crosses among Europeans, Africans and Amerindians. This three-hybrid genetic admixture has been demonstrated by genetic and historical studies (Callegari-Jacques and Salzano, 1999; Alves-Silva *et al.*, 2000; Carvalho-Silva *et al.*, 2001; Mendes-Junior and Simões, 2001; Callegari-Jacques *et al.*, 2003; Barcelos *et al.*, 2006a,b; Suarez-Kurtz *et al.*, 2007; Godinho *et al.*, 2008). Haptoglobin is one of the genetic markers used to describe the genetic constitution of populations. However, there are few data on the haptoglobin gene subtypes in the general Brazilian population, although several reports on Hp subtypes (phenotypes) and types have been published, mostly for indigenous populations (reviewed by Salzano and Callegari-Jacques, 1988; Simões *et al.*, 1989; Salzano *et al.*, 1991, 1997a,b, 1998; Santos *et al.*, 1998; Beiguelman *et al.*, 2003; Calderoni *et al.*, 2006; Zaccariotto *et al.*, 2006). The aim of this work was to examine the frequencies of the Hp gene subtypes in three Brazilian population groups of different ethnic origins (Kayabi Amerindians, Kalunga Afro-descendents and inhabitants of the Federal District).

## Subjects and Methods

###  Populations

1) The Kayabi are a Tupi-Guarani Amerindian tribe (Rodrigues, 1994) with a population of about 1,000 found mainly in the Xingu Indigenous National Park (Mato Grosso state). The Kayabi village sampled consisted of 110 individuals living on the margins of the Teles Pires River (11° 37' 0" S and 55° 40' 60' W) (Dórea *et al.*, 2005; Klautau-Guimarães *et al.*, 2005a,b). More details about this tribe can be found in Dórea *et al.* (2005). The sample (n = 56) used here was collected in 2000 and consisted of 29 males and 27 females, with a median age of 24.5 years and no first-degree (parent-offspring) relationship.

2) The Kalunga are an Afro-derived Brazilian group with an estimated population of 5,300. This group lives in midwestern Brazil, in a rural area of northeastern Goiás State (15° 30' S to 16° 03' S ; 47° 25' W to 48° 12' W) (Oliveira *et al.*, 2002). Historically and numerically, the Kalunga are one of the most important Brazilian Afro-derived populations known as quilombos. The Kalunga are organized into several subregions with different degrees of isolation. The sample (n = 70) used here was collected in 2001 and 2002 and consisted of 29 males and 41 females from the Vão das Almas and Vão de Muleque subregions, with a median age of 42.9 years and a relationship coefficient of up to 1/16.

3) The Federal District (15° 30' S to 16° 03' S and 47° 25' W to 48° 12' W) was founded in 1960 and in 2007 had an urban population of 2,455,903 (2007 IBGE census). Most of the Federal District population initially consisted of migrants from other regions of Brazil (Queiroz 2006), and currently almost half of the Districts inhabitants are migrants (www.distritofederal.df.gov.br). The sample used here (n = 132) was collected in 2002 and consisted of 54 males and 78 females and with a median age of 21.1 years. Based on the subjects self-declared skin color, 68.5% were racially mixed, 24.7% were white, 1.7% was black and 5.1% did not declare their color.

In each of the communities sampled, the aims of this study were explained and the voluntary nature of the donation of biological material was emphasized. Informed consent was obtained in all cases and oral informed consent was obtained in the Kayabi village.

###  Haptoglobin (Hp) genotyping

About 5 mL of peripheral blood was collected by venipuncture using Vacutainer tubes with EDTA as anticoagulant, and then cooled as quickly as possible. DNA was isolated from the buffy-coat layer by using a purification kit GFX (GE Healthcare, Buckinghamshire, England) and the samples were stored below –20 °C until analysis.

Hp genotypes were determined by allele-specific PCR (polymerase chain reaction) as described by Yano *et al.* (1998). The identification of alleles Hp^*1F^, Hp^*1S^ and Hp^*2^ was based on three independent PCR reaction product analyses. PCR products were separated by electrophoresis in 6% polyacrylamide gels under non-denaturing conditions and then detected by staining with silver nitrate.

###  Data management and statistical analysis

Allelic and genotypic frequencies were estimated by gene counting and the goodness of fit of the genotype distribution to the Hardy-Weinberg equilibrium was assessed by the chi-square (χ^2^) test. Values of p > 0.05 indicated Hardy-Weinberg equilibrium. Haptoglobin genetic diversity was assessed by comparing the observed and expected heterozygosities and the F-statistics. Probability (p) values for heterozygote excess were generated by the Genepopweb statistical program version 3.4 (http://genepop.curtin.edu.au/). Comparisons between the different ethnic groups (heterogeneity test) were based on contingency tables analyzed by χ^2^ tests.

## Results

Table 1 summarizes the distribution of the Hp allele frequencies in the populations studied. The Hp^*1^ allele frequency varied from 58.6% in the Afro-descendant Kalunga population to 43.7% in the indigenous Kayabi population. Based on the F-statistics (Table 1), the Brazilian population showed low genetic differentiation among subpopulations (F_ST_) for Hp polymorphism, despite the large variation in Hp^*1F^ frequencies.

Table 2 shows the Hp genotype frequencies in the Kayabi, Kalunga and Federal District populations and the results of the Hardy-Weinberg equilibrium test. The genotypic distributions indicated Hardy-Weinberg equilibrium, except for the Kayabi population, in which there was a heterozygote excess (F_IS_ = -0.5876), as shown in Table 1. Heterogeneity tests for all pairwise comparisons among the three populations revealed significant differences (Table 2).

## Discussion

In this study, there were significant differences in Hp allele frequencies among the three Brazilian populations of different ethnicities. The Hp^*1^ allele frequency was higher in the Kalunga (Afrodescendents) and lower in the Kayabi (Amerindian), in which the Hp^*2^ allele was most frequent. These results agreed with literature (reviewed by Carter and Worwood 2007), since they showed geographical differences in the Hp^*1^ and Hp^*2^ frequencies, depending on the ethnic origin of the group. We also observed significant differences in the distribution of the Hp^*1F^ and Hp^*1S^ alleles. The results for the Kalunga, Kayabi and Federal District agree, respectively, with the Hp^*1^ allele frequencies described for Brazilian Afro-descendants (55%) (Tondo *et al.*, 1963), Içana River Indians (43%) (Simões *et al.*, 1989) and an urban group from São Paulo (46.5%) (Wobeto *et al.*, 2007). The Hp^*1^ allele frequency in the Kalunga (58%) was also similar to that described for the African continent (0.56) and North America (0.55), but higher than in Asia (0.27) (Carter and Worwood, 2007), probably because of the contribution of Afro-descendants that formed the quilombo. For the Kayabi, the frequency of the Hp^*1^ allele (44%) was similar to that reported for three South American Amerindian populations, viz., the Makiritare (42%), Kubenkokre (49%) (Arends *et al.*, 1970; Santos *et al.*, 1998) and the French Guiana population of Kaliña (44.5%) (Mazières *et al.*, 2007), but was lower than for other Brazilian Amerindians (Salzano *et al.*, 1974, 1991, 1997a,b, 1998; Oliveira *et al.*, 1998).

The Hp^*1^ allele frequency (49.6%) of the Federal District urban population was similar to that reported for other Brazilian urban populations from São Paulo state (46%) (Wobeto *et al.*, 2007) and Euro-descendants from Porto Alegre (41.4%) (Tondo *et al.*, 1963). The distribution of the Hp alleles in the Federal District population probably reflects the history of the creation of Brasília, the new Brazilian capital, in the late 1950s. Unlike most Brazilian cities, Brasília and the accompanying Federal District were completely new projects in which settlement of the Federal Capital was driven by government benefits. The construction of Brasília (1956-1960) was the main attraction for migrants, who came from northern, southeastern and southern Brazil (Queiroz, 2006). As a result, the population of Brasília and the Federal District was formed by a wide-ranging mixture of migrants from all regions of Brazil (Queiroz, 2006) that reflected five centuries of interethnic crosses among people of European, African and Amerindian descent (Alves-Silva *et al.*, 2000; Carvalho-Silva *et al.*, 2001; Mendes-Junior and Simões, 2001; Vargas *et al.*, 2006; Suarez-Kurtz *et al.*, 2007; Godinho *et al.*, 2008). This very diverse origin of the Federal District population has made it the most representative sample-group of the Brazilian population.

The present study provides the first report of variations in the Hp^*1F^ and Hp^*1S^ frequencies in non-Amerindian Brazilians. The frequency of the Hp^*1F^ allele was highest in the Afro-descendant Kalunga (29.3%) and lowest in the indigenous population of Kayabi (2.6%). This finding agrees with reports in which a higher Hp^*1^ frequency has been linked to a higher Hp^*1F^ frequency (Delanghe *et al.*, 2000; Carter and Worwood, 2007). In addition, the Hp frequency distribution in the populations studied was not homogenous (p < 0.0001 in the heterogeneity test) and probably reflected selection or recent ethnic admixture. The Hp^*1F^/Hp^*1S^ allele frequency ratios among these populations were 0.06 for the Kayabi, 1.0 for the Kalunga and 0.26 for the Federal District. This variation was attributable essentially to the Hp^*1F^ allele since variation in the Hp^*1S^ allele frequency was very low among these populations (no marked geographical differences). Similar variation in the Hp^*1^ allele has been reported for Central American populations (Delanghe *et al.*, 2000), although the ratios were different from those seen here.

The Kayabi population had Hp genotype frequencies that were not in Hardy-Weinberg equilibrium, with an excess of heterozygotes (F_IS_ = -0.5876). Factors that could account for this finding include the following: (i) the Kayabi live in an area that has experienced intense migration as a result of gold prospecting, and they have consequently become somewhat mixed (Klautau-Guimarães *et al.*, 2005b), (ii) the Hp phenotypes are associated with several disorders such as diabetes and cardiovascular and infectious diseases (Langlois and Delanghe, 1996; Sadrzadeh and Bozorgmehr, 2004; Carter and Worwood, 2007) that may have subjected the population to some form of natural selection and (iii) the Kayabi population consumes freshwater fish contaminated by monomethyl mercury, and is also exposed to endemic infectious diseases such as malaria, for which they lack basic medical services (Dórea *et al.*, 2005, Klautau-Guimarães *et al.*, 2005a); the latter two hypotheses suggest selection in favor of heterozygotes.

Since F_IT_ and F_IS_ represent the correlations between the two uniting gametes that produce individuals in the total population and subpopulations, respectively (Nei 1977), and since our results for these parameters were negative in both cases, this implies an excess of heterozygotes in both situations. However, these results were affected by the Kayabi population. In addition, given that F_ST_ is the correlation between two gametes drawn at random from each subpopulation and measures the degree of genetic differentiation of subpopulations (Nei 1977), our results (F_ST_ = 0.0291) showed that there was a slight genetic differentiation among subpopulations of the general Brazilian population studied.

In conclusion, we have provided the first description of the distribution frequencies of the Hp subtypes Hp^*1F^ and Hp^*1S^ in three Brazilian populations of different ethnic origins. For haptoglobin polymorphism, despite the large variation in Hp^*1F^ frequencies, results of F_ST_ (0.0291) indicated slight genetic differentiation among subpopulations of the general Brazilian population studied. This polymorphism has proven to be an interesting biomarker for understanding human migrations around the world and for identifying associations with diseases. Additional studies are required to map the distribution of these Hp subtypes in other ethnicities, and to gain a better understanding of the biological significance of this marker for anthropogenic studies.

## Figures and Tables

**Table 1:** Distribution of haptoglobin allele frequencies and F-statistics in the Kayabi, Kalunga and Federal District populations.

Populations	Hp^*1^ alleles			Hp^*2^ allele	Heterozygosity observed (H_i_)	Heterozygosity expected (H_s_)	F_IS_ (inbreeding coefficient)	${\bar{F}}_{IS}$	F_ST_	F_IT_
	Hp^*1S^	Hp^*1F^	Total							
Kayabi (n = 56)	0.411	0.026	0.437	0.563	0.8214*	0.5174*	-0.5876	-0.1592	0.0291	-0.1255
Kalunga (n = 70)	0.293	0.293	0.586	0.414	0.7429	0.6570	-0.1308			
Federal District (n = 132)	0.394	0.102	0.496	0.504	0.4697	0.5800	0.1902			
Mean	0.366	0.140	-	0.494	0.6779	0.5849				

*p < 0.05. P-values data for heterozygote excess were generated using statistical program Genepopweb version 3.4.

**Table 2:** Frequencies of the haptoglobin genotypes and the Hardy-Weinberg (HW) and heterogeneity test in the Kayabi, Kalunga and Federal District populations.

Populations	Genotypes		N	Genotype frequencies	HW test (p-values)	Heterogeneity test (p-values)
Kayabi (n = 56)	1-1	Hp^*1S^Hp^*1S^	0	0	0.000	Kayabi x Kalunga < 0.0001
		Hp^*1S^Hp^*1F^	3	0.05		
		Hp^*1F^Hp^*1F^	0	0		
			
	2-1	Hp^*1S^Hp^*2^	43	0.77		
		Hp^*1F^Hp^*2^	0	0		
			
	2-2	Hp^*2^Hp^*2^	10	0.18		

Kalunga (n = 70)	1-1	Hp^*1S^Hp^*1S^	6	0.09	0.130	Kalunga x Federal District < 0.0001
		Hp^*1S^Hp^*1F^	14	0.20		
		Hp^*1F^Hp^*1F^	2	0.03		
			
	2-1	Hp^*1S^Hp^*2^	15	0.21		
		Hp^*1F^Hp^*2^	23	0.33		
			
	2-2	Hp^*2^Hp^*2^	10	0.14		

Federal District (n = 132)	1-1	Hp^*1S^Hp^*1S^	26	0.20	0.051	Federal District x Kayabi < 0.0001
		Hp^*1S^Hp^*1F^	13	0.10		
		Hp^*1F^Hp^*1F^	2	0.02		
			
	2-1	Hp^*1S^Hp^*2^	39	0.30		
		Hp^*1F^Hp^*2^	10	0.08		
			
	2-2	Hp^*2^Hp^*2^	42	0.30		
